# Clarifying photoluminescence decay dynamics of self-assembled quantum dots

**DOI:** 10.1038/s41598-019-41075-7

**Published:** 2019-03-15

**Authors:** Minh Tan Man, Hong Seok Lee

**Affiliations:** 1 Institute of Theoretical and Applied Research (ITAR), Duy Tan University, Hanoi, 100000 Vietnam; 2 Department of Physics, Research Institute of Physics and Chemistry, Chonbuk National University, Jeonju, 54896 Republic of Korea

## Abstract

We studied the temperature-dependent photoluminescence (PL) and time-resolved PL spectra of multilayer CdTe/ZnTe quantum dots (QDs) to understand their carrier dynamics. We demonstrated a method of enhancing the confinement of carriers in CdTe QDs by modulating the number of stacked layers, leading to enhanced acoustic phonons up to 67 μeV and reducing the optical phonon coupling to 20 meV with an average phonon energy of 20 meV. The temperature-dependent decay time could be explained using a simple model of the thermal redistribution of carrier states. Thermal escape from hole states during multiphonon scattering occurred only at high temperatures, whereas blue shifts and enhanced PL intensity were expected to enhance the electron–phonon coupling and confinement-induced mixing among discrete state and continuum states with separation energies of 3.5–7.4 meV. Time-resolved PL measurements probed the electric field screening effect as a function of the strain distribution in QDs and was established to be 2.5 ± 0.2 MV/cm.

## Introduction

Semiconductor quantum dots (QDs) that display a large nonlinear optical response, ultrafast signal switching, quantum efficiency, and high-temperature stability are important for use in future photonic devices, optical data storage, and optical computing^1–4^. Self-assembled CdSe/ZnSe QDs are a particularly promising single photon emitter system^5–7^. Self-assembled QDs form as a consequence of layer-to-layer strain relaxation, which often results in non-uniform particles and difficulties controlling dot size and density^8–10^. These synthetic difficulties highlight important issues around controlling the size, density, and uniformity of the particles because the optical properties of QD-based photonic devices depend on the size, density, and uniformity of QDs. Control over the growth parameters, reorganization of a surface into islands through post-growth thermal annealing, the presence of a capping layer, and the effects of different substrates can successfully manipulate the size, density, and uniformity of QDs^11–13^. Previous work explained carrier dynamics using the simple model that includes discrete transitions and the escape of carriers in nonradiative processes^14^. However, a full assignment of the spectral response and blue-shifted because of electric field screening effect remains unexplored. In particular, intermixing effects produced by the layer-by-layer assembly of QDs, which enhances electron–hole wave function overlap, provide an additional tool for understanding the carrier dynamics and relaxation processes in quantum-confined nanostructures.

Detailed knowledge of the carrier dynamics, the existence of a larger valence band offset, and intermixing effects between QDs and separation layers in multilayer CdTe/ZnTe QDs on Ga substrates are crucial issues in the fabrication photonic devices. In the present study, multilayer CdTe/ZnTe QDs on a GaAs substrate, in which the QDs were prepared with various CdTe stacked layer numbers, were fabricated using molecular beam epitaxy (MBE) and atomic layer epitaxy (ALE) methods. We measured the temperature-dependent and time-resolved PL properties of all samples. Exciton coupling to both acoustic and optical phonons was investigated. This study revealed a method of further enhancing the confinement of carriers in CdTe QDs, enhancing the acoustic phonon up to 67 μeV. The optical phonon coupling was reduced to 20 meV with an average phonon energy of 20 meV. Temperature-dependent and time-resolved PL studies were carried out to study the thermal redistribution of electrons and holes over discrete states. Although nonradiative processes affect carrier relaxation via thermal escape involving hole states, the energy separation among the fine-structured states was expected to differ in the stacked layers for a number of reasons: (a) electron–phonon coupling was enhanced; (b) confinement-induced mixing occurred between discrete states and continuum states due to the intermixing among layers of the QDs and separation layers.

## Results

### Energy separation and optical phonon scattering

**Figure 1:**
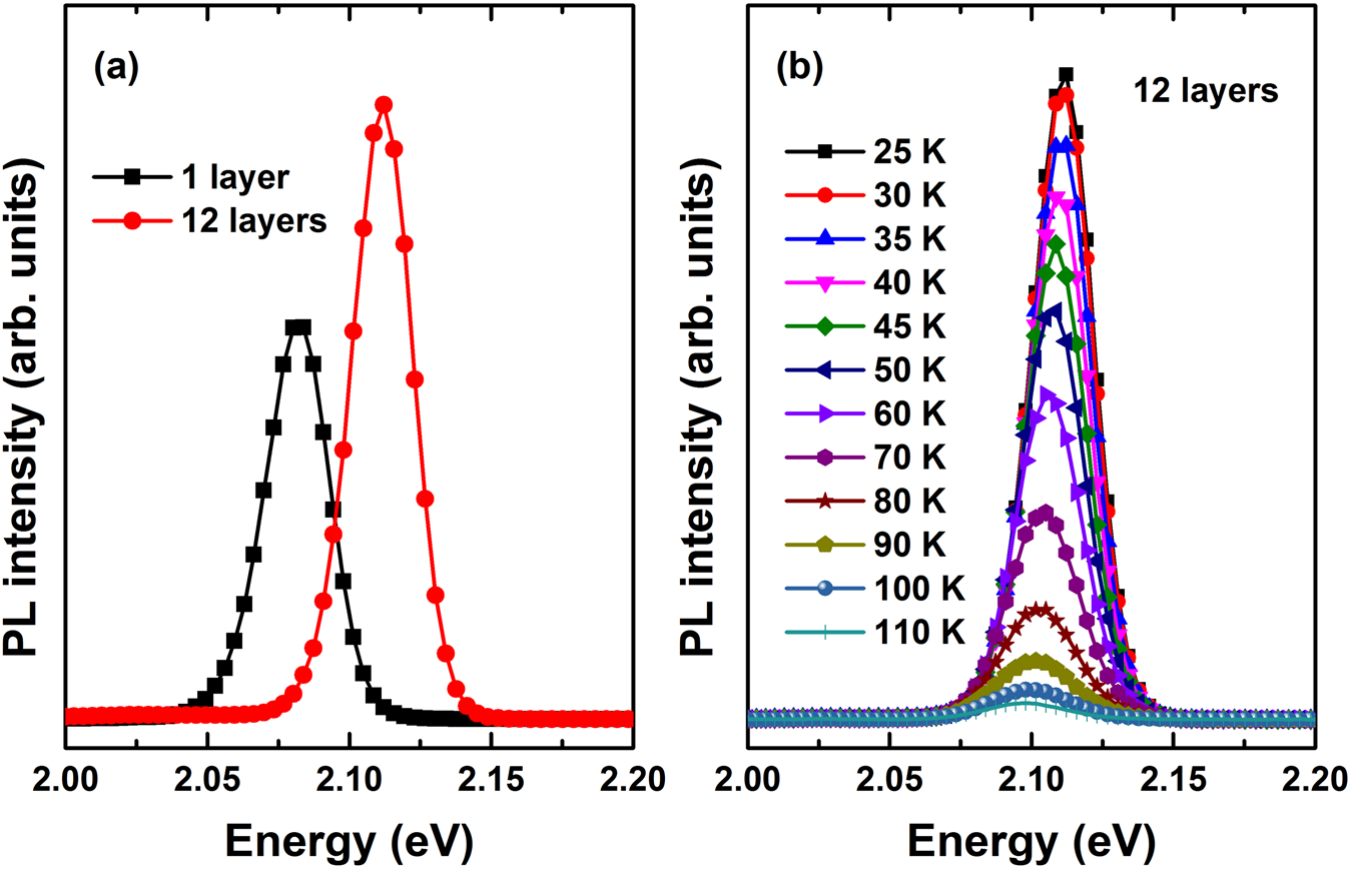
(**a**) PL spectra at 25 K for the multilayer CdTe/ZnTe QDs prepared with 1 and 12 layers. (**b**) PL spectra at several temperatures for the 12 layer sample.

**Figure 2:**
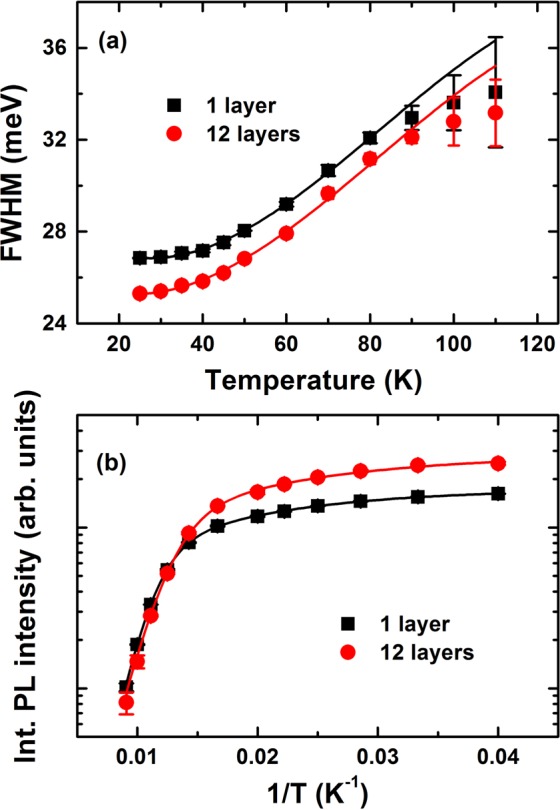
(**a**) Measured (symbols) and calculated (solid lines) FWHM; and (**b**) Integrated PL intensities as a function of the reciprocal temperature for the stacked CdTe/ZnTe QDs. The line shows the best fit curve using the model described in the text.

Figure 1(a) shows representative PL spectra of all samples investigated at 25 K under low excitation power. These curves clearly revealed that the PL transition shifted to higher energies with increasing numbers of stacked layers. The quantum confinement effect and thermally-induced carrier redistribution^14,15^ in samples with a greater number of stacked layers enhanced the PL intensity and also reduced the full width at half maximum (FWHM) from 26.9 meV in a single layer to 25.3 meV in 12 stacked layers. As shown in Fig. 1(b), the PL maximum shifted to lower energies at higher temperatures, reaching 2.08 and 2.11 eV for 1 layer and 12 layers, respectively. The temperature-dependent PL spectra shifts may have been caused by inhomogeneities^16^, localized carrier escape, diffusion to traps, and intermixing effects among the assembled QDs^17–20^. The exciton linewidth Γ(*T*) at any temperature could be fit to the following equation^21^ [Fig. 2(a)], Γ(T) = *σ*_*LA*_*T* + Γ_*LO*_*N*_*LO*_(*T*). Inhomogeneities were characterized by measuring the inhomogeneous broadening, which revealed Γ_0_ to be 26.9 or 25.3 meV for 1 or 12 stacked layers samples, respectively [Fig. 2(a)]. Reductions in inhomogeneous broadening were always caused by fluctuations and the intermixing effects of the assemble QDs, suggesting that spacer layers permitted strain relaxation, leading to QDs that clustered and reduced the multilayers’ inhomogeneous broadening. Localized carrier escape was characterized using the exciton–acoustic phonon coupling coefficient, *σ*_*LA*_, which played a dominant role at low temperatures relative to the energy separation between the difference states. This coupling coefficient was calculated to be 34 or 67 μeV/K (0.72 μeV for bulk CdTe^22^) in the 1 or 12 stacked layer samples, respectively. The third item, Γ_*LO*_, was characterized by calculating the exciton–optical (LO) phonon coupling, and ${N}_{LO}(T)={[\exp ({E}_{LO}/{k}_{B}T)-1]}^{-1}$ indicated the number of phonons with an average energy (*E*_*LO*_). We obtained Γ_*LO*_ values of 20 meV for both samples. The values of *E*_*LO*_ were 19.3 and 20 meV for 1 and 12 stacked layer samples, respectively. As the number of layers increased, the PL intensity increased and shifted to higher energies, suggesting that the QDs were clustered, thereby enhancing the intermixing layers and the surface roughness during the layer-by-layer assembly of QDs. These effects decreased inhomogeneous broadening and enhanced exciton–acoustic phonon coupling due to separation energy at low temperatures.

We examined whether the temperature-dependent PL intensity arose from the energy separation between the different states or the thermal escape process. As shown in Fig. 2(b), the integrated PL intensities as a function of temperature for confined carriers in stacked QDs suggested that the main process could be explained based on the redistribution of carriers in localization potentials^14^. The thermally induced integrated PL intensities could be modeled according to the equation

1 $${I}_{PL}(T)=\frac{{I}_{0}}{1+a\,\exp (\,-\,{\rm{\Delta }}E/{k}_{B}T)+b\,\exp (\,-\,2{\rm{\Delta }}E/{k}_{B}T)+c{[\exp ({E}_{LO}/{k}_{B}T)-1]}^{-m}},$$

where *a*, *b*, and *c* are constants relating to the energy density of states and *I*_0_ is the integrated PL intensity at 0 K. At low temperatures, the energy separation between the two excited states at thermal equilibrium is given by Δ*E*^14^. The main nonradiative process at high temperatures is a thermal escape process defined by *E*_escape_ = m*E_*LO*_. Here, *m* is the number of LO phonons, and the average energy phonon, *E*_*LO*_, was extracted from the temperature dependence of Γ_*LO*_. As shown in Fig. 2(b), the best fit curve excellently reproduced the integrated PL intensity data for Δ*E* = 4.5 and 7.3 meV, and *E*_escape_ = 48.25 and 60 meV for 1 and 12 stacked layer samples, respectively. The increase in both Δ*E* and *E*_escape_ in the 12 stacked layers provided further evidence for the enhanced confinement of excitons in the QDs, which were explained by in-plane carrier transfer between weakly-localized QDs connected by clustering, surface roughness, and the presence of wetting layers. Moreover, the Δ*E* values extracted from this temperature-dependent PL intensity were consistent with coupling between the 1 S[1S_3/2_-1S_e_], 2 S[2S_3/2_-1S_e_] and 1 P[1P_3/2_-1P_e_] states and confined acoustic phonons, yielding discrete recombination and thermal redistribution among exciton states.

### PL decay dynamics

**Figure 3:**
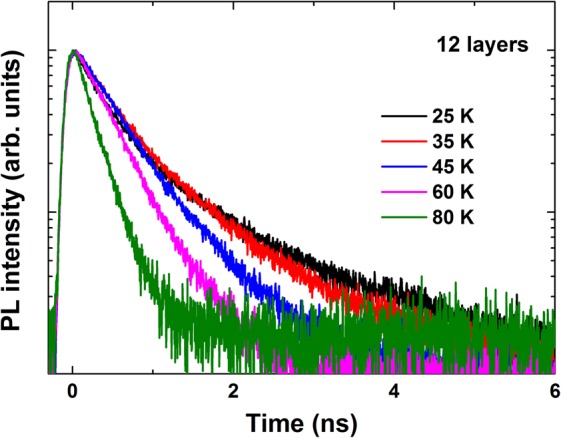
Time-resolved PL spectra at several temperatures for the 12 layer sample.

**Figure 4:**
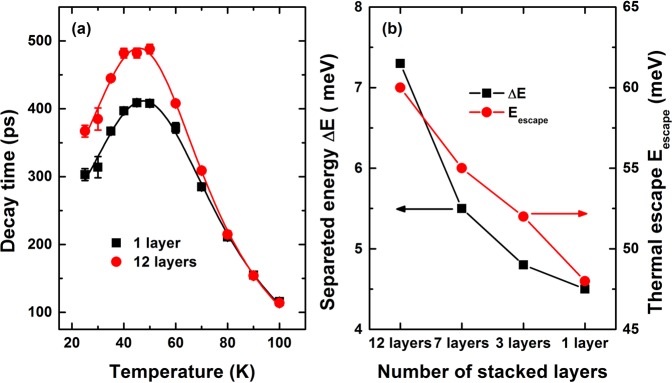
(**a**) Measured (symbols) and calculated (solid lines) decay times as a function of temperature, in samples having 1 layer (*τ*_*s*_ = 325 ps, *τ*_*p*_ = 64 ps, Δ*E* = 4.5 meV), 12 layers (*τ*_*s*_ = 403 ps, *τ*_*p*_ = 52 ps, Δ*E* = 7.3 meV). (**b**) The separation energy Δ*E*, and thermal escape energy *E*_escape_ as a function of the number of stacked dot layers.

Figure 3 shows the time-resolved PL spectra of 12 stacked layers at various temperatures up to 80 K. The decay times extracted from the 1 and 12 layer samples are shown in Fig. 4(a). The characteristic decay times increased slightly below 35 K and remained almost unchanged between 35 and 55 K, whereas they decreased sharply at higher temperatures. For temperatures below 60 K, the decay time evolved in a manner that paralleled the redistribution of localized states, characterized by discrete recombination. Fluctuations in the separation layers may have induced different charge densities and configurations among the carriers surrounding the QDs, and these effects could play an essential role in the carrier redistribution process. Thus, the QD exciton decay time at low temperatures was essentially radiative. At high temperatures, a single exponential decay was observed. Excitons were delocalized into the QDs, and nonradiative processes were dominated by a thermal escape process, drastically reducing their decay times. The temperature-dependent decay times of these processes could be described by^14^

2 $$\frac{1}{\tau }=\sum \frac{1}{{\tau }_{i}}P(i)=\frac{p(s)}{{\tau }_{S}}+\frac{p({\rm{\Delta }}E)}{{\tau }_{p}}+\frac{1}{{\tau }_{esc}}$$

The levels involved were the ground state, which achieved s states with decay times *τ*_*s*_, whereas the *p* states displayed a decay time *τ*_*p*_, shorter than the decay time *τ*_s_. $p(s)=\frac{2}{Z(T)}$ and $p({\rm{\Delta }}E)=\frac{2}{Z(T)}\exp (\,-\,{\rm{\Delta }}E/{k}_{B}T)$ indicated the occupation probabilities of the s and p orbitals, which depended on the separation energy Δ*E*. The partition function *Z*(*T*) versus temperature was given by, $Z(T)=2+4\exp (\,-\,{\rm{\Delta }}E/{k}_{B}T)+6\exp (\,-\,2{\rm{\Delta }}E/{k}_{B}T)$^14^. The thermal escape rate, $1/{\tau }_{esc}$, estimated the energy difference between the 1 S(h) and 2 S(h) hole states, which resulted in the 1st exciton 1 S(h)-1S(e) and the 2nd exciton 2 S(h)-1S(e) transitions^23–25^ and was given by $1/{\tau }_{esc}={{\rm{\Gamma }}}_{LO}{[\exp ({E}_{LO}/{k}_{B}T)-1]}^{-m}$.

The value of *τ*_*s*_ was obtained only when the *s*-shell was occupied at temperatures of a few Kelvin. The other parameters were extracted from the experimental data according to Eq. (2), as shown in Fig. 4(a) for the 1 and 12 stacked layer samples as well as for the 3 and 7 layer samples (data not shown). This temperature-dependent behavior was similar to that reported previously for CdTe multilayer QDs on Si substrates. Interestingly, the best fit values were very similar to the values extracted from the integrated PL intensity analysis. The Δ*E* and *E*_*escape*_ values clearly increased as a function of the number of stacked layers, and the extracted Δ*E* fell in the range 4.5 < Δ*E* < 7.3 meV [Fig. 4(b)]. We found that the excited state decay dominated the response by the discrete *s*-states, whereas the thermal populations of the *p*-states were negligible at 25 K. At higher temperatures, the 1 P electron states began to be populated, which prohibited the *s*-*p* transitions, leading to an increase in the decay time. This process continued to dominate the response until the two states were nearly equally populated at 55 K. The onset of this process was determined by the separation energy Δ*E*. The decay time *τ*_*p*_ was determined to be 64–52 ps. Above 55 K, the main thermal escape process increased in prominence due to scattering with *m* (2.1 and 3) LO-phonons, clearly suggesting that relaxation into hole states (1S_3/2_, 2S_3/2_, and 1P_3/2_) due to the first occupation occurred much faster than the radiative decay time.

## Discussion

**Figure 5:**
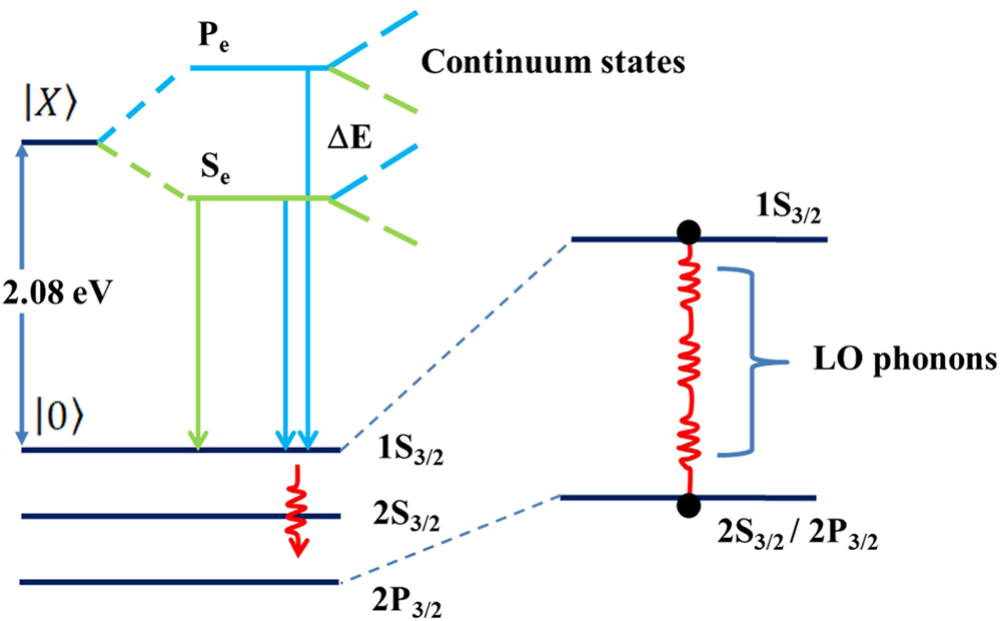
Schematic illustration of the different contributions to the discrete state, continuum states, and thermal escape in self-assembled CdTe QDs.

**Figure 6:**
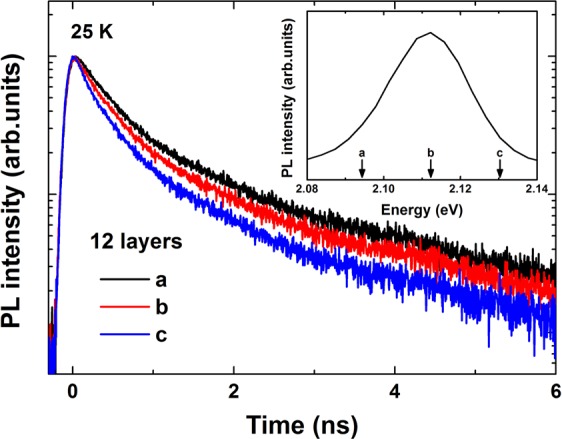
Time-resolved PL curves collected from stacked CdTe/ZnTe QDs prepared with CdTe QDs having 12 layers at 25 K for various detection energies.

**Figure 7:**
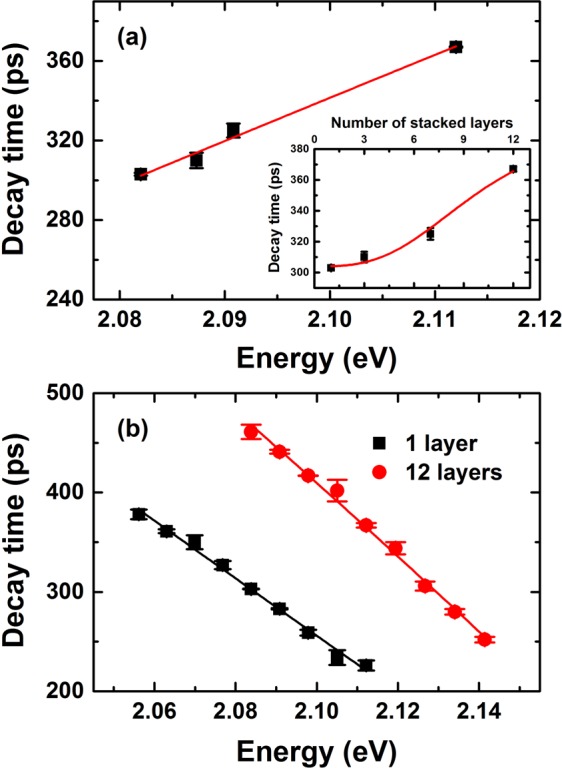
(**a**) Measured decay time (symbol) versus measured energy of the PL peak for the samples with different numbers of stacked layers. Inset: Measured (symbols) and calculated (solid lines) decay times for localized excitons in continuum states in the intermixing layers. (**b**) Detection energy dependence of the decay times in the QDs samples. Solid lines show the best fit curves under an effective electric field of 2.5 ± 0.2 MV/cm in the intermixing layers.

Currently, questions remain as to whether the separation energy Δ*E* in the QDs depended primarily on thermally activated transfer from the dark exciton state to the bright exciton state with an activation energy of a few meV, resulting in a PL blue shift and an enhanced PL intensity of QDs. This is a well-known effect observed during the growth of the dot radius^26^. The separation energy has been attributed to a variety of causes. Transitions between intrinsic and surface states^27^, an increase in the electron–hole pairs due to surface-trapped carriers^28,29^ and the thicknesses of the ZnTe separation layers do not affect the quantum barrier resulting from the growth of defects in CdTe QDs with a large number of layers. The separation energy Δ*E* also corresponds to the energy of a confined acoustic phonon in QDs^30–32^. Moreover, it has been shown that at low temperatures it is necessary to take into account the fact that in quantum dots PL originates mainly from localized states and its temperature dependence exhibits S-shape like behavior, and the interplay of homogeneous versus inhomogeneous broadening^33,34^. In our experiments, such behavior has previously been seen in CdTe QDs on Si substrates^14^, the average number of e-h pairs per QD, is bout 0.1–1 pairs, which is small enough to neglect Auger scattering, and continuum band shifting due to bandgap renormalization. We took advantage of the fact that in QDs, the dark–bright exciton transitions are strongly size-dependent, whereas the separation energy Δ*E* and energy of the confined acoustic phonon depend predominantly on the overall size (i.e., shell, continuum layer, or wetting layer). We therefore suggested that the continuum states of the intermixing layers formed during the layer-by-layer assembly of QDs, which has been characterized by other groups using AFM studies^19,20,35,36^. The continuum density of states in the intermixing layers is given by $\frac{{m}^{\ast }{L}_{x,y}^{2}}{\pi {\hslash }^{2}}$, where ${L}_{x,y}^{2}$ is the typical area accessible to a continuum state wave function and m* is the electron/hole mass. According to the diagram in Fig. 5, similar decay times were expected for recombination into discrete states, consistent with the overlap between carrier wave functions having the same symmetry. This model perfectly reproduced the increase in decay times on the 20–55 K side. The thermal redistribution of electron and holes over discrete states and continuum states suggested that the blue shifts (28 meV) and enhanced PL intensity (48%) were attributed to processes such as the enhancement of electron–phonon coupling and confinement-induced mixing between discrete states and continuum states. Figure 6 shows time-resolved PL curves taken at different detection energies after pulsed excitation of the initial photon fluence. *j*_p_, equal to 0.75 × 10^11^ photons cm^−2^ per pulse, corresponded to the average number of e-h pairs per QD, about 0.1–1 pairs. A progressive increase in the high-detection wavelength slope of the time-resolved PL at 25 K indicated that continuum bands attributed to e-h pairs created in the intermixing layer relaxed to the QDs. This result clearly demonstrated that the lateral confinement effects dominated other effects. The ZnTe separation layers experienced biaxial compressive strain perpendicular to the area accessible to a continuum state $({L}_{x,y}^{2})$ and the photocreated carriers created an internal electric field that spatially separated the electron and hole wave functions and changed their recombination probability. The lateral confinement effects were sharply enhanced, as shown in Fig. 7(a,b). In any case, the decay time of QDs excitons at low temperatures was essentially radiative, reflecting overlap among the carrier wave functions, and was controlled by the size effects and internal electric field. Our results allowed us to consider the value of the internal electric field or oscillator strength, which were calculated for various numbers of stacked dot layers^37,38^,

3 $$\frac{1}{{\tau }_{rad}}={E}_{p}E{|\langle {{\rm{\Phi }}}_{e}|{{\rm{\Phi }}}_{h}\rangle |}^{2}\frac{{e}^{2}n}{6\pi {\hslash }^{2}{m}_{0}{c}^{3}{\varepsilon }_{0}},$$

where *n* is the CdTe index of refraction and *m*_0_ is the electron mass. *E*_p_ is the Kane matrix element, which characterizes the optical transition in the bulk material and equals 21 eV for an II–VI material^37^. *E* is the energy transition. Φ_*e*_ and Φ_*h*_ are the envelope functions of the electrons and holes. Using Eq. (3), we calculated the electron and hole functions. The corresponding radiative decay times, ${\tau }_{rad}=A/(E/{|I|}^{2})$, are presented in Fig. 7(a,b), where A is the only fit parameter related to the matrix element, and $I\propto |\langle {{\rm{\Phi }}}_{e}|{{\rm{\Phi }}}_{h}\rangle |$ is defined by the overlap integral of the electron and hole envelope functions along the growth direction. A variation in the calculations allowed us to determine the value of 2.5 ± 0.2 MV/cm for an effective electric field in the samples. Decay times in the range of several picoseconds were obtained, and these values increased almost cubically with the stacked dot layer number, as shown in the inset of Fig. 7(a). The value of the internal electric field supported the presence of continuum states, which were screened by e-h pairs in the intermixing layers.

In summary, we used temperature-dependent and time-resolved PL measurements to investigate the number of stacked layers as a factor contributing to the deterioration of the quantum efficiency optical performances in multilayer CdTe/ZnTe QDs on GaAs substrates. We demonstrate that the continuum states of the intermixing layers/wetting layers enhanced carrier confinement in the CdTe QDs. As the result, acoustic phonons up to 67 μeV and optical phonon coupling down to 20 meV with an average phonon energy (E_LO_) of about 20 meV were determined. A thermally activated transition occurred between two different states separated by 3.5–7.4 meV. This transition was attributed to confinement-induced mixing between discrete state and continuum states, whereas thermal escape only involved hole states due to scattering via multiphonons with an average energy of 19–20 meV at high temperatures. We also demonstrated that time-resolved photoluminescence provides a probe of exciton localization in CdTe quantum dots.

## Methods

### Sample structure

The samples were fabricated on the GaAs (100) substrate using MBE and ALE processing. The GaAs substrates were degreased in warm trichloroethylene, cleaned in acetone, cleaned in Br-methanol solution, and thoroughly rinsed in de-ionized water. After this chemical cleaning and drying by nitrogen gas, the GaAs substrates were mounted on the molybdenum susceptor, and then, themally cleaned at 580 °C for 5 min. The vertically stacked CdTe QDs were grown as follow: a 900-nm-thick ZnTe buffer layer was grown on the GaAs substrate using MBE, and then, 4.5 monolayer (ML) CdTe QDs were grown using ALE. A 15-nm-thick ZnTe separation layer was grown on the 4.5 ML CdTe QDs using MBE, and then, 4.5 ML CdTe QDs were grown using ALE. These CdTe/ZnTe layer structure was more repeated several times. Lastly, a 100-nm-thick ZnTe capping layer was grown on the CdTe QDs using MBE. In this work, chosen four samples have CdTe QDs layer of 1, 3, 7 and 12 layers, respectively. The ZnTe and CdTe layers were grown at substrate temperature of 310 °C. The Cd, Zn and Te source temperatures used for growth of the ZnTe and CdTe layers were 195, 280, and 310 °C, respectively.

### Measurement techniques

The PL emission was dispersed using a 150 mm monochromator and was detected using a multichannel plate photomultiplier tube. A 405 nm picosecond laser diode with an 80 MHz repetition rate was used as an excitation source. Temperature-dependent PL spectra were measured using a He closed-cycle Displex refrigeration system, which varied the sample temperature between 25 and 110 K. Time-resolved PL decay curves were measured using a time-correlated single photon counting (TCSPC) method. A commercially available TCSPC module (PicoHarp, PicoQuant GmbH, Berlin, Germany) was used to obtain the PL decay curves. The FWHM of the total instrument response function was less than 130 ps.
